# Carbon ion irradiation exerts antitumor activity by inducing cGAS–STING activation and immune response in prostate cancer‐bearing mice

**DOI:** 10.1002/cam4.6950

**Published:** 2024-02-01

**Authors:** Wei Hu, Zhenshan Zhang, Yushan Xue, Renli Ning, Xiaomao Guo, Yun Sun, Qing Zhang

**Affiliations:** ^1^ Department of Radiation Oncology, Shanghai Proton and Heavy Ion Center Fudan University Cancer Hospital Shanghai China; ^2^ Shanghai Key Laboratory of Radiation Oncology (20dz2261000) Shanghai China; ^3^ Shanghai Engineering Research Center of Proton and Heavy Ion Radiation Therapy Shanghai China; ^4^ Department of Research and Development, Shanghai Proton and Heavy Ion Center Fudan University Cancer Hospital Shanghai China

**Keywords:** carbon ion irradiation, cGAS–STING signaling pathway, immune response, prostate cancer

## Abstract

**Background and Purpose:**

As an advanced radiotherapy technique, carbon ion radiotherapy has demonstrated good efficacy and low toxicity for prostate cancer patients, but the radiobiological mechanism of killing tumor cells has not been fully elucidated. This study aims to explore the antitumor effects of carbon ion irradiation (CIR) through investigating the immune response induced by CIR in prostate cancer‐bearing mice and the underlying molecular mechanism.

**Materials and Methods:**

We established subcutaneous transplantation tumor models of prostate cancer to evaluate the tumor inhibition effect of CIR. Investigation of immunophenotype alterations were assessed by flow cytometry. Immunofluorescence, western blot, and real‐time quantitative PCR was employed to analyze the activation of cGAS–STING pathway.

**Results:**

CIR showed more powerful tumor growth control than photon irradiation in immunocompetent syngeneic C57BL/6 mice. CIR exerts antitumor effect by triggering immune response, characterized by increased CD4^+^ T cells and macrophages in tumor, enhanced CD8^+^ T cells and T effector memory cells in spleen, improved IFN‐γ production of CD8^+^ tumor‐infiltrating lymphocytes, and reduced exhausted T cells in tumor and spleen. Additionally, production of cytoplasmic double‐stranded DNA, protein levels of p‐TBK1 and p‐IRF3 in the cGAS–STING pathway, and gene expression levels of downstream interferon‐stimulated genes were significantly increased after CIR in a dose‐dependent manner. Treatment of RM1 tumor‐bearing mice with the STING inhibitor C‐176 impaired the antitumor effect of CIR.

**Conclusion:**

The excellent antitumor activity of CIR in immunocompetent prostate cancer‐bearing C57BL/6 mice may be attributed to stronger induction of antitumor immune response and higher activation of cGAS–STING pathway.

## 1 INTRODUCTION

As one of the main therapeutic strategies for prostate cancer, radiotherapy have significantly improved treatment outcomes.^1^, ^2^ Nevertheless, reports of the “abscopal effect” of radiation therapy^3^, ^4^ have helped people realize that radiotherapy is far more than local treatment, that is, the underlying immunomodulatory effect makes radiotherapy an integral part of systemic treatment. However, the further increase of radiation dose is limited due to a difficult balance of maximizing tumor control and minimizing normal tissue toxicities. Moreover, the existence of radioresistance might narrow down the efficacy of photon radiotherapy in prostate cancer. Under the circumstances, there is still room for improvement in radiotherapy for prostate cancer.

The efficacy of radiotherapy is heavily dependent upon the body's immune system. For instance, Lee et al.^5^ found that CD8^+^ T cells are critical for local control of radiation therapy, while inhibition of CD8^+^ T cell infiltration in the tumor microenvironment can lead to the occurrence of radiation resistance.^6^ Prostate cancer is manifested as low tumor mutation burden, scarcity of tumor infiltrating CD8^+^ cytotoxic T cells, and infiltration of multiple immunosuppressive cell subsets in the tumor microenvironment,^7^, ^8^, ^9^, ^10^ in which case, resistance to photon radiation therapy is induced through various mechanisms,^11^, ^12^, ^13^ thus compromising the improvement of therapeutic efficacy. The exploration of radiotherapy strategy to improve immune response is an important issue to improve the therapeutic effect of prostate cancer.

Carbon ion radiotherapy is a cutting‐edge radiation therapy technique with physical and biological superiorities, and has become one of the few important clinical therapies that may overcome radiation resistance. The potential advantage of inducing immune response may be a contributing factor for that, either the presence of “Bragg peak” causing less damage to peripheral lymphocytes, or its role in inducing more tumor‐associated antigen release and more effective antitumor immunity.^14^ Up to now, the immunomodulatory effects of photon irradiation (PhIR) have been extensively explored; nevertheless, the effects of carbon ion irradiation (CIR) on immune response is widely illuminated in neither animal models nor patients, especially in prostate cancer. We have previously studied the immune modulation of carbon ion radiotherapy in peripheral blood in patients with localized prostate cancer and demonstrated the potential of carbon ion radiotherapy to elicit immune activation.^15^ In the current study, we investigated the immunologic effects of CIR in the spleen and tumors of prostate cancer‐bearing mice. Furthermore, it is unknown what molecular mechanisms drive the immune response of CIR.

There is increasing evidence that high‐linear energy transfer (high‐LET) ionizing radiation modality, such as CIR, induces more complex and even clustered DNA double strand breaks (DSBs) than low‐LET PhIR.^16^ The cGAS–STING pathway plays a crucial role in linking DNA damage to immune responses. Not surprisingly, CIR‐induced DNA fragments may be smaller and more likely to leak into the cytoplasm than PhIR does, triggering Type I interferon transcription and activating immune response via cGAS–STING pathway.^17^ Higher degree of activation of cGAS–STING signaling pathway may be associated with the immunomodulatory advantage of CIR. However, there is still a lack of relevant experimental data available on this subject.

For these reasons, the effect and mechanism of CIR on the immune response in tumor‐bearing hosts should be elucidated in order to develop more practical strategies in clinical settings. Herein, we evaluated the influence on immune cell infiltration and T‐cell effector function in prostate cancer‐bearing mice to study the immune reaction evoked by CIR and explored the mechanisms underlying CIR‐induced antitumor efficacy, involved in cGAS–STING signaling pathway. The findings are helpful to enrich the radiobiological effects of CIR and of translational significance for optimizing carbon ion therapy strategies for prostate cancer patients.

## 2 MATERIALS AND METHODS

### 2.1 Cell culture

Murine prostate cancer cell line RM1 was purchased from the Chinese Academy of Sciences Cell Bank. The cell line was maintained in RPMI 1640 medium (BasalMedia), supplemented with 10% fetal bovine serum (FBS; Gibco), and 1% penicillin/streptomycin (Gibco) in a humidified atmosphere of 37°C and 5% CO_2_.

### 2.2 Irradiation

PhIR was performed by x‐ray beams (225 kV, 13.3 mA, 40 cm SSD) filtered with 2 mm Al generated by a PXi Precision X‐RAD 225 at a dose rate of 3.198 Gy/min. CIR was performed using a heavy ion synchrotron accelerator (Siemens AG) at Shanghai Proton and Heavy Ion Center (SPHIC). In brief, CIR was delivered with a homogeneous spread‐out Bragg peak with the energy of 156.3 MeV/u (dose averaged LET 50 keV/μm) on the target. For in vitro experiments, T25 cell culture flasks (Corning) were placed at a tailored vertical stand oriented perpendicular to the carbon ion beam. For in vivo experiments, the prostate cancer‐bearing mice were anesthetized by intraperitoneal injection of pentobarbital sodium (50 mg/kg) and affixed to a platform perpendicular to the beam line. A customized protection device was adopted to make sure that only the tumor area was exposed to the field of radiation.

### 2.3 Mouse models and treatments

To establish tumor models, 1 × 10^6^ RM1 cells were implanted subcutaneously in the right hind flank of syngeneic C57BL/6 mice or BALB/c nude mice. Irradiation commenced on Day 10 after tumor inoculation when the mean tumor volume reached about 80–100 mm^3^. For C‐176 experiment, C‐176 (S6575, Selleck; 5 mg/kg/d) was intraperitoneally injected into mice daily from 7 days before irradiation to the day of radiation therapy. Tumor size was measured by digital calipers every 2 days and tumor volume was calculated as length × width^2^/2 (mm^3^). The ethical tumor volume limit in this study was set at 1000 mm^3^. Mice were sacrificed and tissues were analyzed at a specified time. All animal experiments were approved by the SPHIC Institutional Animal Care and Use Committee.

### 2.4 Flow cytometry

Mice were sacrificed by cervical dislocation, and spleens and tumors were harvested to prepare single cell suspensions. The back of syringes was used to dissociate spleens and red blood cell lysing buffer (BD Pharmingen) was used to lyse erythrocytes. Tumors were minced with scissors, then digested with 1 mg/mL Collagenase D (Roche) and 0.2 mg/mL DNase I (Roche) dissolved in Hank's Balanced Salt Solution using the gentleMACS Octo Dissociator with Heaters (Miltenyi Biotec) equipped with gentleMACS C Tubes (Miltenyi Biotec). To exclude dead cells, the cell suspensions were stained with Fixable Viability Stain 780 (BD Pharmingen) for 10 min at room temperature. Cell surface markers were assessed using the following antibodies incubated with cell suspensions in the dark for half an hour at 4°C: CD45 V500 (BD Pharmingen), CD3 PE (BD Pharmingen), CD4 BV605 (BD Pharmingen), CD8 FITC (BD Pharmingen), CD44 PE‐Cy7 (BD Pharmingen), CD62L PerCP‐Cy5.5 (BD Pharmingen), PD1 BV421 (BioLegend), CD11b APC (BioLegend), and F4/80 PE (BioLegend). For analysis of intracellular cytokine, Leukocyte Activation Cocktail with GolgiPlug (BD Pharmingen) was used to stimulate cells for about 5 h before staining. After incubation with Fixable Viability Stain 780 and surface antibodies successively, followed by permeabilization with Cytofix/Cytoperm™ Fixation/Permeablization Kit (BD Pharmingen), cells were stained with IFN‐γ APC (BD Pharmingen) for 30 min at 4°C and then washed twice. Flow cytometry was performed using CytoFLEX S (Beckman Coulter) and analyzed with CytExpert (Beckman Coulter).

### 2.5 Statistical analysis

The data were analyzed using GraphPad Prism version 7.0.0 and SPSS software Version 20.0. Statistical analysis between two groups was performed using two‐tailed unpaired Student's *t*‐test. Comparisons of multiple groups were performed using one‐way analysis of variance (ANOVA). Tumor volumes were analyzed using two‐way ANOVA. *p* Value < 0.05 was considered statistically significant. Significance was shown as *p < 0.05, **p < 0.01, ***p < 0.001, and ****p < 0.0001.

For colony formation assay, cell viability assay, cell apoptosis assessment, immunofluorescence, immunohistochemistry, western blot, and quantitative real‐time PCR, see the Data S1.

## 3 RESULTS

### 3.1 CIR suppresses tumor growth of tumor‐bearing mice

**FIGURE 1:**
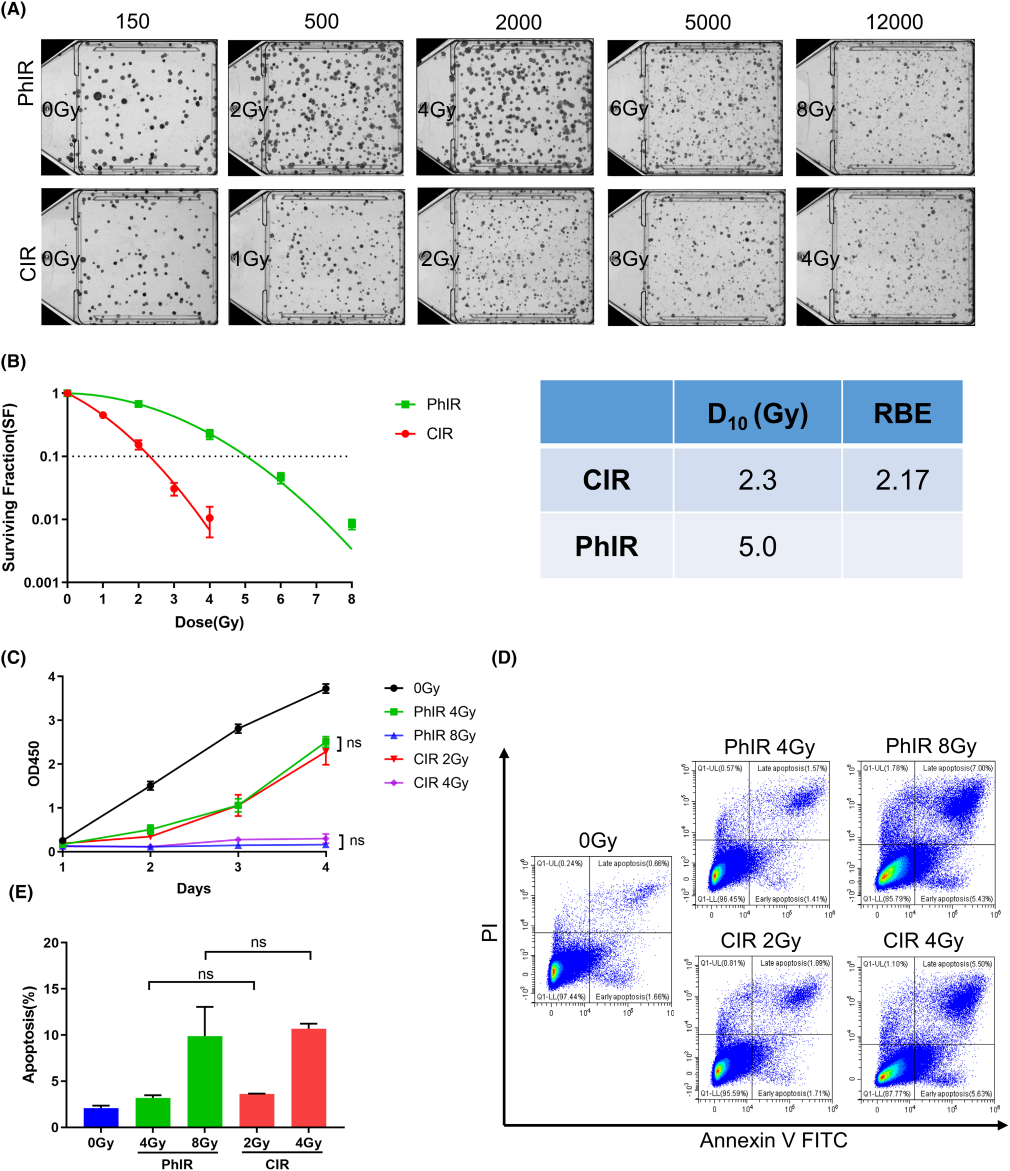
Carbon ion irradiation (CIR) exhibits higher relative biological effect. (A) Scanning photos of colonies of RM1 cells irradiated with different doses of photon irradiation (PhIR) or CIR in colony formation assay. (B) Left, surviving fraction of RM1 cells irradiated with PhIR or CIR. Right, irradiation dose of 10% survival rate (D10) and RBE of CIR versus PhIR. (C) Cell proliferation of RM1 cells was analyzed by CCK‐8 assay after PhIR and CIR. (D) Cell apoptosis of RM1 cells was measured by flow cytometry with Annexin V FITC/PI staining after PhIR and CIR. (E) Bar graph showing the proportion of apoptotic cells. Data represent the mean ± SD of three independent experiments for (B), (C) and (E). ns, not significant, by two‐tailed unpaired Student's *t*‐test (C and E).

To compare PhIR and CIR at biological equivalent dose (BED), colony formation assay was conducted to determine that the relative biological effectiveness (RBE) of CIR at the 10% survival level compared with PhIR in RM1 cells was 2.17 (Figure 1A,B). The reliability of the RBE measurement was supported by similar dose‐dependent decreases in cell proliferation rate (Figure 1C) and increases in cell apoptosis rate (Figure 1D,E) after BEDs of CIR and PhIR. Then we choose the BED of 5 Gy(RBE) for animal experiments, which means that 2.5 Gy CIR is approximately equal to 5 Gy PhIR. This dose was selected based on previous research^18^ and our pilot study, which required appropriate control of the tumor growth rate while leaving tissue available for assessment.

**FIGURE 2:**
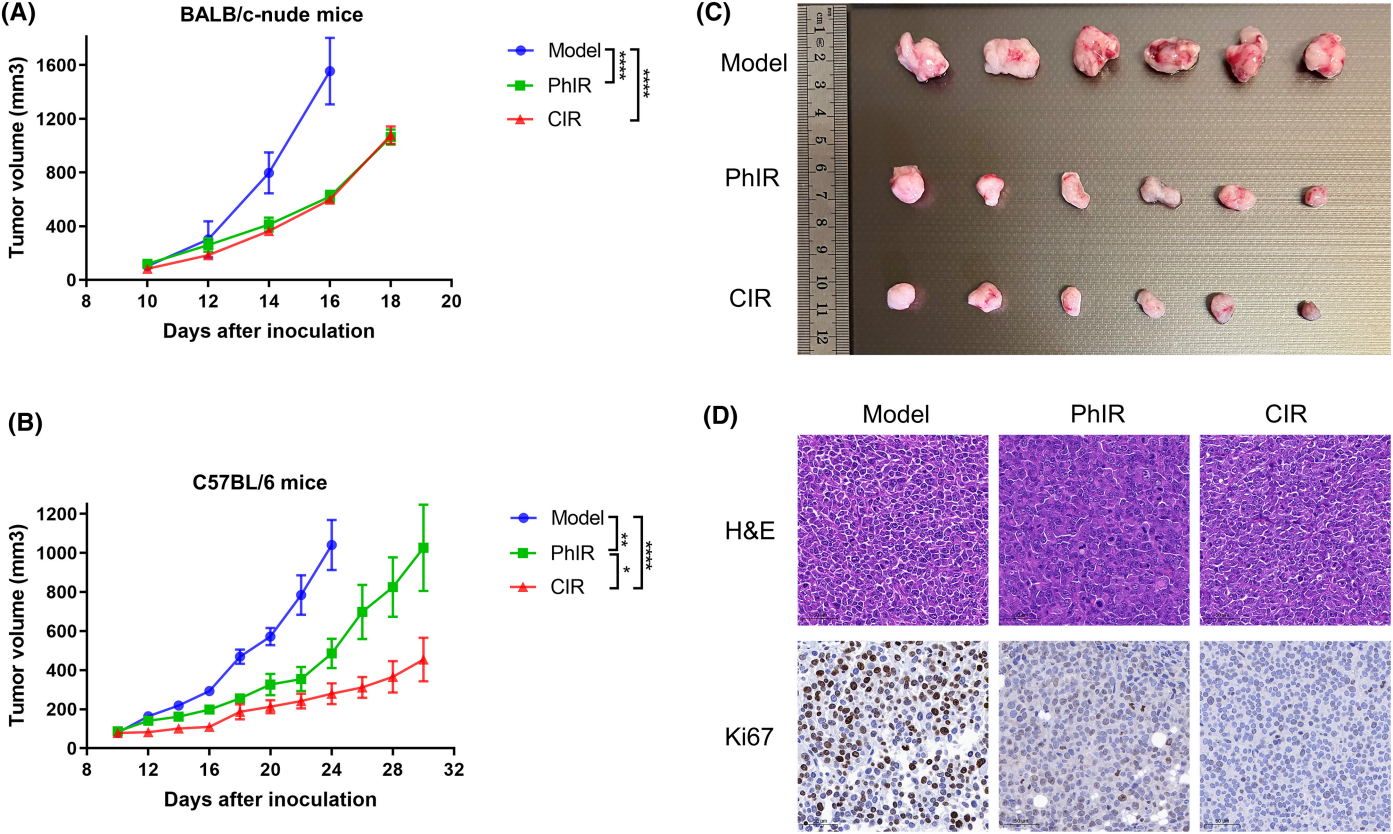
Carbon ion irradiation inhibits (CIR) tumor growth. Tumor volume (mm^3^) for (A) BALB/c nude mice and (B, C) C57BL/6 mice inoculated with 1 × 10^6^ RM1 cells (Model) and treated with CIR and photon irradiation (PhIR) on Day 10 after inoculation. *n* = 6 mice/group. (D) Representative images of H&E and immunohistochemistry staining of Ki67. Scale bar, 50 μm. Data represent the mean ± SEM. *p < 0.05, **p < 0.01, and ****p < 0.0001, by two‐way analysis of variance.

We first attempted to investigate the effect of CIR and PhIR on tumor growth in RM1 tumor‐bearing immunodeficient BALB/c nude mice. Our results indicated that tumor growth was significantly reduced at similar rate after CIR and PhIR compared to unirradiated mice (Figure 2A). However, when RM1 cells were inoculated into immunocompetent syngeneic C57BL/6 mice, the CIR group showed more powerful tumor growth control than the PhIR group (Figure 2B,C). Furthermore, CIR significantly decreased the expression of Ki67 (Figure 2D). These surprising results suggested that the observed stronger tumor‐suppressive effect of CIR over PhIR may be related to an intact immune system.

### 3.2 CIR induces cytoplasmic DNA and cGAS–STING activation, and is functionally responsible for the observed tumor growth inhibition

**FIGURE 3:**
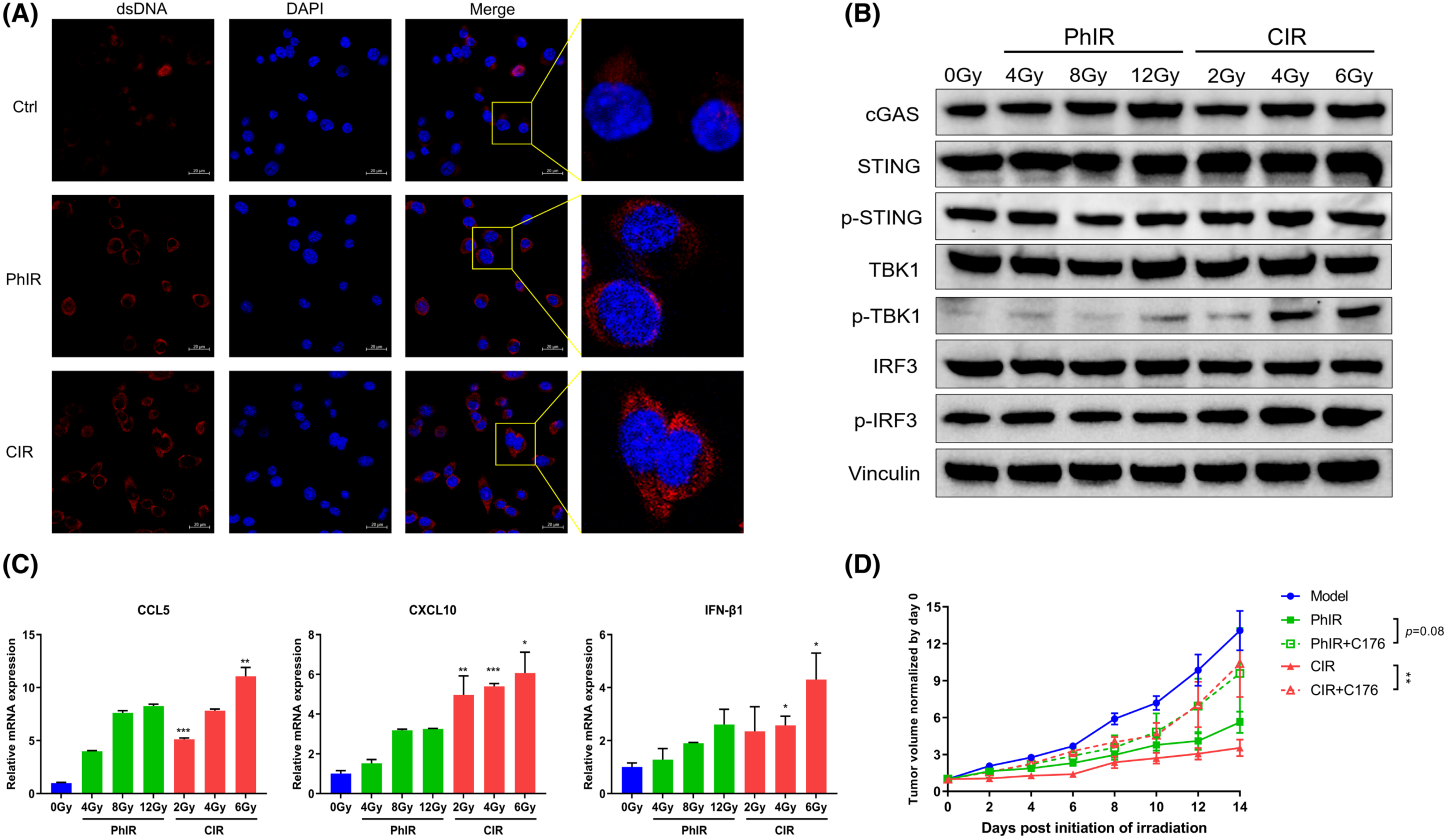
Carbon ion irradiation (CIR) promotes antitumor effect via increasing the cytoplasmic release of dsDNA and activating cGAS–STING pathway. (A) Immunofluorescence assay was used to determine the dsDNA accumulation in cytoplasm of RM1 cells irradiated with photon irradiation (PhIR) or CIR. The representative images were captured using confocal microscope. Scale bar: 20 μm. (B) Western blot analysis of a few key factors of cGAS–STING pathway in RM1 cells irradiated with different doses of PhIR and CIR. (C) The mRNA levels of cGAS–STING downstream molecules (CCL5, CXCL10 and IFN‐β1) were analyzed by qRT‐PCR in RM1 cells treated with PhIR and CIR. PhIR was compared with CIR of the corresponding BED. These experiments were repeated 3 times. Data represent the mean ± SD. (D) Tumor volume (mm^3^) for RM1 tumor‐bearing C57BL/6 mice treated with PhIR, PhIR+C‐176, CIR and CIR+C‐176. *n* = 6 mice/group. Data represent the mean ± SEM. *p < 0.05, **p < 0.01, and ***p < 0.001, by two‐tailed unpaired Student's *t*‐test (C) or two‐way analysis of variance (D).

We next investigated the molecular mechanisms involved in CIR‐mediated tumor growth suppression. cGAS–STING signaling pathway plays a crucial part in the immune reaction caused by DNA damage. Previous studies^19^, ^20^ revealed that DNA damage induced by radiation therapy activated cGAS–STING signaling pathway, which then mediated immune activation and stimulated antitumor immunity. We thus detected the cytoplasmic dsDNA with confocal microscopy and illustrated that CIR generated significantly higher level of this cGAS–STING activator compared with PhIR and unirradiated control (Figure 3A). Western blot (WB) was performed to analyze the several key proteins involved in cGAS–STING pathway and the results showed higher induction of p‐TBK1 and p‐IRF3 after CIR at BED, indicating activation of this pathway (Figure 3B). Moreover, qRT‐PCR revealed that CIR markedly increased the production of the key downstream molecules CCL5, CXCL10, and IFNβ1 in cGAS–STING pathway, while a modest advantage of CIR over PhIR was shown (Figure 3C). Furthermore, intraperitoneal injection of the STING inhibitor C‐176 in RM1 tumor‐bearing mice weakened the antitumor activity of CIR and showed a tendency to impair antitumor effect of PhIR (Figure 3D). Taken together, we believed that CIR could activate cGAS–STING signaling pathway and be responsible for the inhibition of tumor growth in prostate cancer‐bearing mice.

### 3.3 CIR exerts antitumor effect by triggering immune response

**FIGURE 4:**
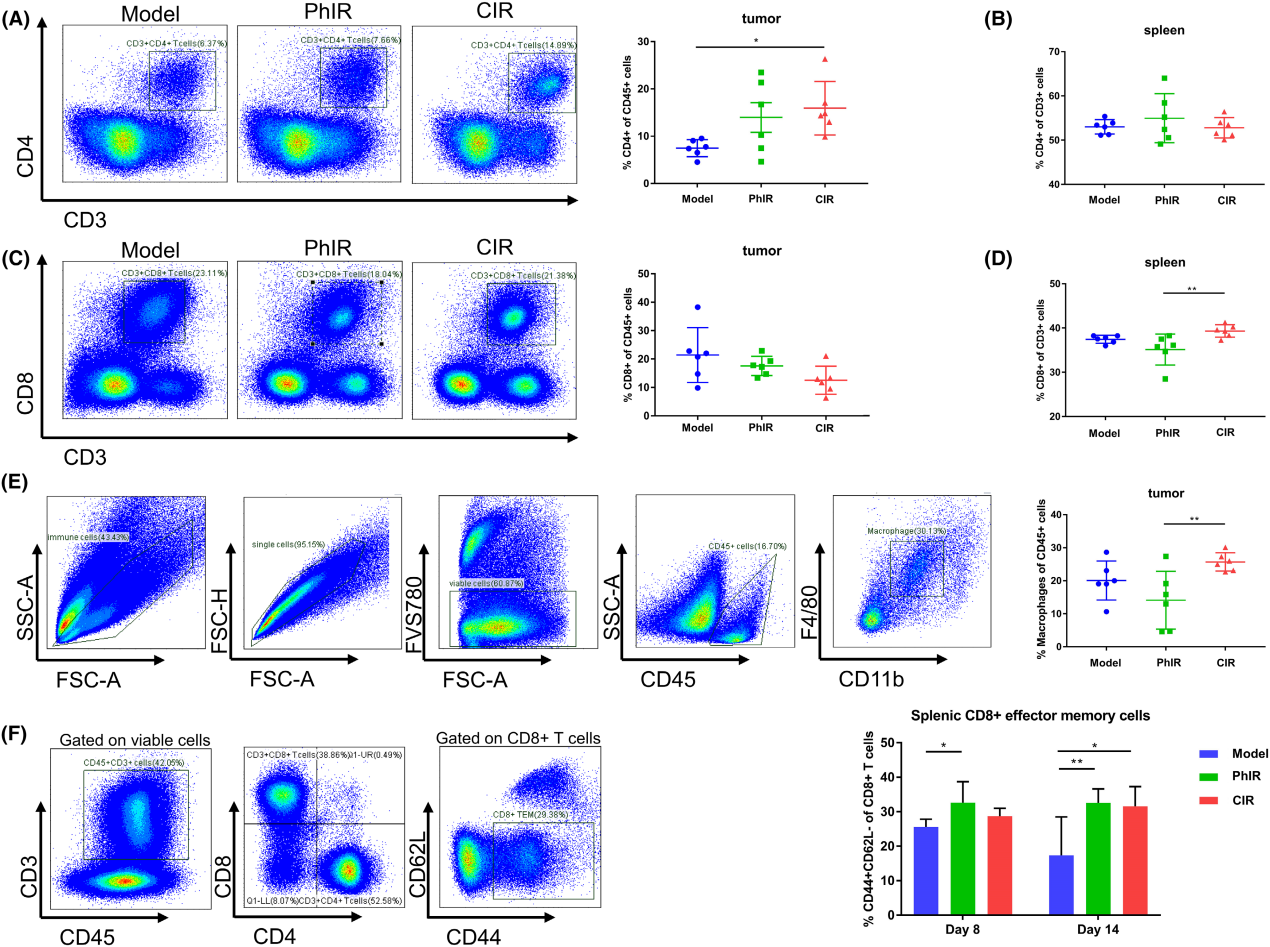
Carbon ion irradiation (CIR) boosts antitumor immune responses in RM1 tumor‐bearing C57BL/6 mice. (A) Left, representative flow plots of CD4^+^ tumor‐infiltrating lymphocytes (TILs) on Day 8 after irradiation for C57BL/6 mice inoculated with 1 × 10^6^ RM1 cells (Model) and treated with photon irradiation (PhIR) and CIR. Right, quantification of the percentages of CD4^+^ TILs on Day 8 post‐irradiation. (B) Quantification of the percentages of CD4^+^ T cells in spleen on Day 8 post‐irradiation. (C) Left, representative flow plots of CD8^+^ TILs on Day 8 post‐irradiation. Right, quantification of the percentages of CD8^+^ TILs on Day 8 after irradiation. (D) Quantification of the percentages of CD8^+^ T cells in spleen on Day 8 post‐irradiation. (E) Left, gating strategy of macrophages in the tumor is shown. Right, quantification of the percentages of intratumoral macrophages on Day 8 after irradiation. (F) Left, gating strategy of CD8^+^ T effector memory cells in spleen is shown. Right, quantification of the percentages of splenic CD8^+^ TEM on Day 8 and Day 14 after irradiation. *n* = 6 mice/group. Data represent the mean ± SD. *p < 0.05 and **p < 0.01, by one‐way analysis of variance.

The above results indicated that under normal immune conditions, CIR exhibited a more significant therapeutic effect than PhIR, which suggested that CIR might be more capable of immunomodulatory effects. We first examined CD4^+^ and CD8^+^ T cells in the tumor and spleen on Day 8 post‐irradiation, and representative gating for CD4^+^ and CD8^+^ tumor‐infiltrating lymphocytes (TIL) was shown (Figure 4A,C). There was a significant increase of CD4 TILs in CIR‐exposed mice compared with the nonirradiated tumor‐bearing mice, while the percentages of CD4^+^ T cells in spleen showed no significant difference (Figure 4A,B). Although the level of CD8^+^ TILs did not change significantly after CIR, CD8^+^ T cells in spleen displayed an increase after CIR compared to PhIR (Figure 4C,D). Similar results were observed for the percentage of macrophages in tumor microenvironment, with significantly more macrophages in tumor microenvironment post‐CIR compared with post‐PhIR (Figure 4E). The changes of CD4^+^ and CD8^+^ T cells were also accompanied by a rise of memory T cells in spleen, that is, CD8^+^ T effector memory cells (TEM) increased significantly in PhIR‐treated mice than unirradiated control on Day 8 after irradiation, while CD8^+^ TEM displayed a significant increase in both CIR and PhIR‐treated mice on Day 14 (Figure 4F). In short, these results suggested that CIR triggered antitumor immune response together with immune memory in syngeneic RM‐1 prostate cancer‐bearing mice.

### 3.4 CIR promotes functionally competent CD8 ^+^ T cells

**FIGURE 5:**
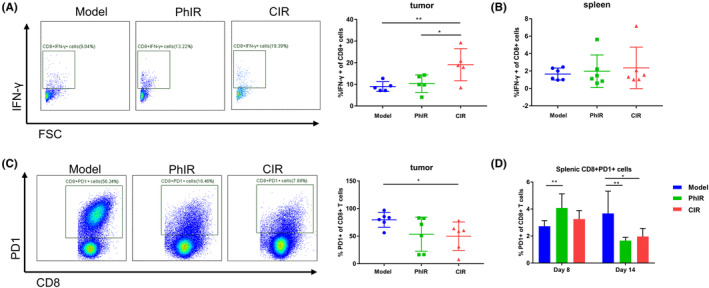
Carbon ion irradiation (CIR) improves CD8^+^ T cell effector function and attenuates CD8^+^ T cell exhaustion marker PD1 expression in RM1 syngeneic prostate cancer‐bearing C57BL/6 mice. (A) Left, representative flow plots of CD8^+^ IFN‐γ^+^ tumor‐infiltrating lymphocytes (TILs) on Day 8 after irradiation for C57BL/6 mice inoculated with 1 × 10^6^ RM1 cells (Model) and treated with photon irradiation (PhIR) and CIR. Right, quantification of the percentages of CD8^+^ IFN‐γ^+^ TILs on Day 8 after irradiation. (B) Quantification of the percentages of CD8^+^ IFN‐γ^+^ T cells in spleen on Day 8 after irradiation. (C) Left, representative flow plots of CD8^+^ PD1^+^ TILs on Day 8 after irradiation. Right, quantification of the percentages of CD8^+^ PD1^+^ TILs on Day 8 after irradiation. (D) Quantification of CD8^+^ PD1^+^ T cell percentages in spleen on Day 8 and Day 14 after irradiation. *n* = 5–6 mice/group. Data represent the mean ± SD. *p < 0.05 and **p < 0.01, by one‐way analysis of variance.

To explore whether CIR can enhance effector function of CD8^+^ T cells, we first evaluated cytokine IFN‐γ production of CD8^+^ T cells on Day 8 post‐irradiation by stimulating single cell suspensions with ionomycin and phorbol 12‐myristate 13‐acetate (PMA) for about 5 h. Results showed that CIR dramatically enhanced the ability of CD8^+^ TILs to produce IFN‐γ than PhIR and non‐irradiation did, even though IFN‐γ production of CD8^+^ T cells in spleen remained unchanged in these three groups (Figure 5A,B). We also investigated co‐expression exhaustion marker PD1 of CD8^+^ T cells. At Day 8, compared with unirradiated mice, CIR significantly reduced the percentage of CD8^+^ PD1^+^ TILs (Figure 5C). Surprisingly, PD1 expression of CD8^+^ T cells in spleen increased significantly on Day 8 in PhIR‐exposed mice instead of CIR‐treated ones, but declined markedly on Day 14 after irradiation of CIR and PhIR (Figure 5D). In brief, CIR promoted CD8^+^ T cell effector function in RM1 tumor‐bearing C57BL/6 mice.

## 4 DISCUSSION

Our understanding of the immune response evoked by radiotherapy, including antitumor immunity and immunosuppressive effects, has substantially increased. However, the immunomodulatory effects after CIR and PhIR are not exactly the same, resulting in different antitumor activities. In our study, we learnt that CIR could decrease tumor growth in both RM1 tumor‐bearing immunodeficient BALB/c nude mice and immunocompetent syngeneic C57BL/6 mice, yet it failed to eliminate cancer, probably due to the relatively low dose. Compared with immunodeficient mice, CIR showed a greater tumor control advantage than PhIR in immunocompetent mice, suggesting that CIR was sufficient to boost antitumor immune response to a greater extent. This phenomenon that the local tumor control of CIR at BED was superior to that of PhIR has also been demonstrated in melanoma‐bearing mice,^21^ which is also consistent with the better clinical efficacy of carbon ion radiotherapy in a variety of primary cancers.^22^, ^23^, ^24^

The essence of the killing effect of radiotherapy is the DNA damage of tumor cells, and the cGAS–STING pathway mediates antitumor immunity through sensing cytoplasmic DNA, contributing to radiotherapeutic responses.^25^ We observed in our study that CIR was able to produce more cytoplasmic dsDNA than non‐IR and PhIR, and one explanation for this finding was that carbon ion beams seemed more effective in inducing micronuclei and very small DNA fragments.^17^ Then the cGAS–STING signaling was subsequently greatly activated, as the several key protein factors involved in this pathway and the downstream Type I IFN‐stimulated gene (ISG) expression were highly induced. Du et al.^26^ showed that gene expression signatures and biological processes of an esophageal cancer cell line 6–24 h after CIR were different from those of x‐rays and protons, related to Type I IFN response and innate immune response, which was in parallel to our results, albeit from the perspective of transcriptomics.

The immunomodulatory activities of CIR have been explored in a few in vitro and in vivo studies. For example, CIR could cause further increase of damage‐associated molecular patterns on the surface of various tumor cell lines and induction of immunogenic cell death, just as Huang et al.^27^ suggested that CIR induced a higher level of calreticulin exposure than photon and proton irradiation in the glioma cell lines, and Ran et al.^28^ reported that carbon ion beams increased the exposure of high mobility group box 1 than photons in lung cancer cell lines. Besides, CIR was found to reduce the population of myeloid‐derived suppressor cells and M2‐like macrophages, and improve tumor control and survival of melanoma‐bearing mice and glioma‐bearing mice.^21^, ^29^ In this study, we showed that CIR was capable of increasing the intratumoral infiltration of CD4^+^ T lymphocytes and macrophages in prostate cancer‐bearing mice, whether compared with the unirradiated control or PhIR. Intriguingly, an increase in IFN‐γ production of CD8^+^ TILs and a decrease in PD1 expression of CD8^+^ TILs was noted, although CIR did not raise the abundance of CD8^+^ TILs. Similarly, in the B16 and S91 murine melanoma tumor models, CIR induced significant tumor infiltration of IFN‐γ‐expressing cells, which could be further enhanced when combined with anti‐PD1 immune checkpoint inhibitors.^18^ These data indicated that CIR potentiated the tumor immune microenvironment by inducing CD8^+^ TILs to be reprogrammed into more functionally competent cells, which was responsible for better tumor control in prostate cancer‐bearing mice treated by CIR. Additionally, the increase of splenic CD8^+^ PD1^+^ cells on Day 8 after PhIR was consistent with the study reporting that peripheral CD8^+^ PD1^+^ T cells significantly increased after radiotherapy in responsive prostate cancer patients, with an explanation that PD1 expression was expected to be a potential marker for tumor response.^30^ However, this increase of PD1 expression, an indicator commonly considered as a marker of T‐cell exhaustion,^31^ was transient and significantly declined on Day 14 after treatment of CIR and PhIR. CD8^+^ effector T cells were known to be important in preventing tumor recurrence and metastasis, meaning that an increased amount of CD8^+^ TEM in the spleen can provide better protection against tumor post‐PhIR and CIR.

Nevertheless, our study still has some limitations that need to be taken seriously. More than one cell line should be used to construct a mouse model of prostate cancer to investigate the immune response to CIR to increase the reliability of the study. In addition, it is worth mentioning that the immune environment of mice is quite different from that of humans, so the establishment of organoid models or patient‐derived xenograft models may be more effective in enhancing the credibility of research.

In summary, we concluded that CIR exerts excellent antitumor activity, which may be attributed to the induction of cGAS–STING activation and immune response, characterized by increased immune cell infiltration, improved T cell effector function, and enhanced immune memory. Our findings are expected to enrich the knowledge of the carbon ion radiobiological effects, fill in the blank of research on the immunologic effect and mechanism of antitumor effect induced by CIR, and provide a preliminary basis for optimization of carbon ion therapy strategies for patients with prostate cancer.

## AUTHOR CONTRIBUTIONS

**Wei Hu:** Conceptualization (lead); formal analysis (lead); investigation (lead); writing – original draft (lead). **Zhenshan Zhang:** Formal analysis (equal); investigation (equal). **Yushan Xue:** Investigation (supporting). **Renli Ning:** Supervision (equal). **Xiaomao Guo:** Supervision (supporting). **Yun Sun:** Supervision (equal); validation (lead). **Qing Zhang:** Conceptualization (equal); supervision (lead); writing – original draft (equal).

## FUNDING INFORMATION

The authors declare that no funds were received during the preparation of this manuscript.

## CONFLICT OF INTEREST STATEMENT

The authors declare that they have no competing interests.

## ETHICS STATEMENT

All animal experiments were approved by the Shanghai Proton and Heavy Ion Center Institutional Animal Care and Use Committee.

## CONSENT

Not applicable.

## Supporting information


Data S1.

## Data Availability

All data generated or analyzed during this study are included in this published article and its supplementary information files.
