# Supplementary material for: Carbon ion irradiation exerts antitumor activity by inducing cGAS–STING activation and immune response in prostate cancer‐bearing mice
Source: Cancer Med. 2024 Feb 1;13(2):e6950. doi: 10.1002/cam4.6950 (PMC10832322; doi:10.1002/cam4.6950)
Supplement: Supplementary file 1 — Data S1. [file CAM4-13-e6950-s001.docx]

**Supplementary materials and methods**

**Colony formation assay**

The tumor cells were counted and seeded in T25 cell culture flasks in triplicate at appropriate cell densities. After overnight resting, various doses of PhRT (2, 4, 6, 8 Gy) and CiRT (1, 2, 3, 4 Gy) were delivered to the cells. The plated cells were cultured for 7–9 days at 37 °C and 5% CO_2_. Colonies were washed with DPBS and fixed with 4% paraformaldehyde, then stained with crystal violet. Images of colonies were captured and colonies containing more than 50 cells were counted by a colony counting machine (GelCount, Oxford Optronix, UK). The experiment was repeated 3 times. Surviving fractions were calculated based on the plating efficiency of the unirradiated cells and the data were fitted to the linear quadratic (L-Q) equation. The relative biological effectiveness (RBE) was calculated as the ratio of the dose for 10% survival (D_10_) of PhRT to that of CiRT.

**Cell viability assay**

The Cell Counting Kit-8 (CCK-8) assay was performed to assess cell viability after irradiation. Cells were seeded in 96-well plates with six replicate wells per group at 2 × 10^3^ cells/well. At day 1, 2, 3, 4 after irradiation, the culture medium in the 96-well plates was replaced with a 10:1 mixture of medium and CCK-8 solution (Dojindo). After incubation for 1.5 hours at 37°C, the absorbance value was measured at a wavelength of 450 nm using Cytation 3 (BioTek).

**Cell apoptosis assessment**

Annexin V FITC/PI Apoptosis Detection Kit (BD Pharmingen) was used to assess cell apoptosis after irradiation. Cells were trypsinized, washed twice with cold DPBS, and stained with 5 μL Annexin V FITC and 5 μL PI for 15 min at room temperature in the dark. All samples were acquired using CytoFLEX S (Beckman Coulter) and results were analyzed using CytExpert (Beckman Coulter).

**Immunofluorescence**

Cells on 20mm glass bottom dishes were fixed with 4% paraformaldehyde for 10 minutes, and then permeabilized with 0.5% Triton X-100 (Beyotime Biotechnology) for 10 minutes at room temperature. After washing, the cells were blocked with 5% goat serum (Beyotime Biotechnology) for 30 minutes. This was followed by incubation with primary anti-dsDNA antibody (MAB1293, Sigma-Aldrich) overnight at 4°C, and then with Alexa Fluor 594 conjugated goat anti-mouse IgG (H+L) antibody (ab150116, Abcam) for 1 hours at room temperature. Cells were washed and counterstained with DAPI (Beyotime Biotechnology) to visualize the nuclei. Immunofluorescence images were acquired with a confocal laser scanning microscope (Zeiss LSM 800).

**Histological analysis**

Sections were obtained from paraffin-embedded tumor tissues from sacrificed C57BL/6 mice. Then, the sections were stained with hematoxylin/eosin (H&E) according to standard protocols. Immunohistochemistry (IHC) was performed according to the manufacturers' protocols. Briefly, the sections were heated, deparaffinized, rehydrated and placed in sodium citrate buffer (pH 6.0) for antigen retrieval, and the endogenous peroxidase activity was blocked with 3% hydrogen peroxide. The slides were blocked with 3% BSA and incubated with primary antibody against Ki67 (Abcam) at 4 °C overnight, followed by incubation with secondary antibody for 1 hours at room temperature. Next, the sections were stained with DAB detection kit under a microscope and counterstained with hematoxylin. Finally, neutral balsam was used to seal the slides with a cover slip, and typical images were obtained.

**Western blot**

Briefly, cells were lysed in RIPA lysis buffer supplemented with protease inhibitor cocktail and phosphatase inhibitor cocktail (Epizyme), and the total protein concentration was determined by BCA Protein Assay Kit (Epizyme). The cell lysates were mixed with 5× loading buffer and heated for 10 minutes at 100°C. The protein lysates were loaded and separated by SDS-PAGE, and then transferred to PVDF membranes (Millipore). The membranes were blocked in 5% BSA in TBST for 2 hours at room temperature and incubated with the primary antibodies against cGAS (Cell Signaling Technology), STING (Cell Signaling Technology), Phospho-STING (Cell Signaling Technology), TBK1 (Cell Signaling Technology), Phospho-TBK1 (Cell Signaling Technology), IRF-3 (Cell Signaling Technology), Phospho-IRF-3 (Cell Signaling Technology), and Vinculin (Cell Signaling Technology) overnight at 4 °C. Membranes were washed three times and incubated with HRP-linked secondary antibody (Cell Signaling Technology) for 1 hour at room temperature. Chemiluminescence images were acquired and analyzed with Image Lab software.

**Quantitative** **real-time PCR (qRT-PCR)**

Total RNA was isolated from irradiated cells using SteadyPure Quick RNA Extraction Kit (Accurate Biology) according to the manufacturer's protocol and quantified using a NanoDrop Lite spectrophotometer (Thermo Fisher Scientific). cDNA was prepared from 1 µg RNA using Evo M-MLV Mix Kit with gDNA Clean for qPCR (Accurate Biology) and used for quantitative real-time PCR using SYBR Green Premix Pro Taq HS qPCR Kit (Rox Plus) (Accurate Biology) by QuantStudio™ 5 Real-Time PCR System (Applied Biosystems). Primer sequences of different genes were as follows: GAPDH forward, 5′- ACCCAGAAGACTGTGGATGG -3′; reverse, 5′- ACACATTGGGGGTAGGAAC -3′. CCL5 forward, 5′- ATATGGCTCGGACACCACTC -3′; reverse 5′- TCCTTCGAGTGACAAACACG -3′. CXCL10 forward, 5′- CCAAGTGCTGCCGTCATTTTC -3′; reverse, 5′- GGCTCGCAGGGATGATTTCAA -3′. IFN-β1 forward, 5′- CCAGCTCCAAGAAAGGACGA -3′; reverse 5′- CGCCCTGTAGGTGAGGTTGAT -3′. The relative mRNA level was normalized by the expression of GAPDH. The relative expression of each gene was calculated by the 2^−ΔΔCT^ method.
